# BMI in childhood and adolescence is associated with impaired reproductive function—a population-based cohort study from birth to age 50 years

**DOI:** 10.1093/humrep/deab164

**Published:** 2021-08-07

**Authors:** J Laru, R Nedelec, E Koivuaho, M Ojaniemi, M -R Järvelin, J S Tapanainen, S Franks, M Tolvanen, T T Piltonen, S Sebert, L Morin-Papunen

**Affiliations:** 1 Department of Obstetrics and Gynaecology, University of Oulu and Oulu University Hospital, Medical Research Center, PEDEGO Research Unit, Oulu, Finland; 2 Center for Life Course Health Research, University of Oulu, Oulu, Finland; 3 Department of Children and Adolescents, University of Oulu and Oulu University Hospital, Medical Research Center, PEDEGO Research Unit, Oulu, Finland; 4 Department of Life Sciences, College of Health and Life Sciences, Brunel University London, London, UK; 5 Unit of Primary Health Care, Oulu University Hospital, Oulu, Finland; 6 Department of Epidemiology and Biostatistics, MRC-PHE Centre for Environment and Health, School of Public Health, Imperial College London, London, UK; 7 Department of Obstetrics and Gynecology, University of Helsinki and Helsinki University Hospital, Helsinki, Finland; 8 Institute of Reproductive and Developmental Biology, Imperial College London, London, UK

**Keywords:** obesity, overweight, underweight, childlessness, infertility, growth, childhood, adolescence, reproductive function

## Abstract

**STUDY QUESTION:**

What is the association between childhood and adolescent BMI and reproductive capacity in women?

**SUMMARY ANSWER:**

Adolescent girls with obesity had an increased risk of infertility and childlessness in adulthood independently of their marital status or the presence of polycystic ovary syndrome (PCOS).

**WHAT IS KNOWN ALREADY:**

Girls with obesity (BMI (kg/m^2^)>95th percentile) more often exhibit menstrual irregularities and infertility problems as compared to those with normal weight, and premenarcheal girls with obesity have an increased risk of childlessness and infertility in adulthood. Follow-up studies on the relation between childhood and adolescence growth patterns and fertility or parity throughout the reproductive life span are limited.

**STUDY DESIGN, SIZE, DURATION:**

A prospective, population-based cohort study (the Northern Finland birth cohort 1966) was performed with 5889 women born in 1966 and followed from birth to age 50 years. Postal questionnaires at ages 31 and 46 years addressed questions on reproductive capacity evaluated by decreased fecundability, need for infertility assessment and treatment by 46 years of age. Childlessness and number of children by age 50 years were recovered from registers. Women who did not report ever having attempted to achieve pregnancy (n = 1507) were excluded. The final study population included 4382 women who attempted to achieve pregnancy before age 46 years.

**PARTICIPANTS/MATERIALS, SETTING, METHODS:**

Data on BMI were collected by trained personnel at all stages. We assessed association with both prospectively measured BMI at various time points and with early adiposity phenotypes derived from linear mixed models including the timing and the BMI at adiposity peak (AP) and adiposity rebound (AR). Self-reported infertility assessments and treatments were assessed at ages 31 and 46 years. Data on deliveries were collected from the national birth register. Decreased fecundability was defined at age 31 years as time to achieve pregnancy over 12 months. Logistic regression analyses were conducted with adjustments for marital status, education level and smoking at age 31 years. Women with PCOS were excluded from stratification-based sensitivity analyses. Obesity at a specific age group was defined by having at least one BMI value above the 95th percentile during the related period.

**MAIN RESULTS AND THE ROLE OF CHANCE:**

BMI at the age of AR (5–7 years) was not associated with fertility outcomes after adjustments, but girls with AR <5.1 years had a higher risk of remaining childless compared to girls with AR over 5.1 years (adjusted odds ratio (OR): 1.45 (1.10–1.92)). At ages 7–10 and 11–15 years, obesity was associated with decreased fecundability (adjusted OR 2.05 (1.26–3.35) and 2.04 (1.21–3.44), respectively) and a lower number of children. At age 11–15 years, both overweight and obesity were associated with a higher risk of childlessness (adjusted OR 1.56 (1.06–2.27), 1.77 (1.02–3.07), respectively), even after excluding women with PCOS. Underweight at age 11–15 years was associated with an increased risk for infertility treatment (adjusted OR 1.55 (1.02–2.36)) and a tendency for an increased risk for infertility assessment (adjusted OR 1.43 (0.97–2.10)) after excluding women with PCOS.

**LIMITATIONS, REASON FOR CAUTION:**

Despite a high participation rate throughout the follow-up, some growth data for children over the different age groups were missing. Infertility outcomes were self-reported. A potential over-diagnosis of obesity may have reduced the significance of the association between childhood obesity and fertility outcomes, and the diagnosis of PCOS was self-reported.

**WIDER IMPLICATIONS OF THE FINDINGS:**

This study supports previous results showing that girls with obesity in late childhood and in adolescence displayed reduced fertility and an increased risk of remaining childless in adulthood, independently of marital history and PCOS in adulthood. These findings corroborate the body of evidence for a causal relation between early adiposity and the reproductive functions in women. We recommend reinforcing the prevention of obesity in school-age girls to reduce the risk of impaired reproductive functions.

**STUDY FUNDING/COMPETING INTEREST(S):**

NFBC1966 received financial support from University of Oulu Grant no. 65354, Oulu University Hospital Grant no. 2/97, 8/97, Ministry of Health and Social Affairs Grant no. 23/251/97, 160/97, 190/97, National Institute for Health and Welfare, Helsinki Grant no. 54121, Regional Institute of Occupational Health, Oulu, Finland Grant no. 50621, 54231. The Finnish Medical Foundation, the North Ostrobothnia Regional Fund, the Academy of Finland (project grants 315921, 104781, 120315, 129269, 1114194, 24300796), Center of Excellence in Complex Disease Genetics and SALVE, the Sigrid Juselius Foundation, Biocenter Oulu, University Hospital Oulu and University of Oulu (75617), Jalmari ja Rauha Ahokkaan säätiö, The Finnish Medical Foundation, Medical Research Center Oulu, National Institute for Health Research (UK). M. R. J., S. S. and R. N. received funding by the Academy of Finland (#268336) and the European Union’s Horizon 2020 research and innovation program (under Grant agreement no. 633595 for the DynaHEALTH action and GA 733206 for LifeCycle). The funders had no role in study design, in the collection, analysis and interpretation of the data, in the writing of the article and in the decision to submit it for publication. The authors have no conflict of interest to disclose.

**TRIAL REGISTRATION NUMBER:**

N/A.

## Introduction

The prevalence of obesity has doubled in developed countries during the last 10 years and is continuously increasing (Perez, 2013). Childhood obesity in particular is a worldwide epidemic, and is associated with increasing long-term health problems (Ward *et al.*, 2017). While adulthood obesity is a well-known risk factor of infertility and childlessness (Nelson and Fleming, 2007; Brewer and Balen, 2010), there are only limited data concerning the association of childhood growth and obesity with fertility and reproductive capacity later in life.

Girls with obesity have more menstrual irregularities and an increased risk of polycystic ovary syndrome (PCOS) (Lake *et al.*, 1997; Ollila *et al.*, 2016; Koivuaho *et al.*, 2019), the most common cause of anovulatory infertility (Nelson and Fleming, 2007; Brewer and Balen, 2010). However, few longitudinal studies have investigated the association between childhood growth and reproductive capacity, and the studies showed diverging results. An inverted J-shaped relation between adolescent BMI and parity in adulthood has been observed (Jokela *et al.*, 2007). In prospective cohort studies, girls with obesity in premenarche displayed an increased risk of childlessness and infertility in adulthood (Lake *et al.*, 1997; Jacobs *et al.*, 2017), but conflicting results concerning adolescence have been published (He *et al.*, 2018). To our knowledge, there are only two studies investigating the relation between obesity before age 9 years and reproductive capacity in adulthood, and their results have been conflicting (Lake *et al.*, 1997; Jacobs *et al.*, 2017). We are not aware of any studies focusing on the association of infancy and early childhood growth with later fertility.

Recent studies evaluating the association between childhood obesity and metabolic disorders in adulthood have also focused on childhood growth trajectory data in addition to age-specific BMI measures. BMI first increases from birth and reaches a maximum at the age of 9 months (adiposity peak (AP)) and decreases thereafter to reach a nadir at the age of 4–6 years (Silverwood *et al.*, 2009; Koyama *et al.*, 2014; Péneau *et al.*, 2016). A later timing and a higher BMI at AP have been associated with childhood obesity. In particular, an increased magnitude of AP has been associated with higher BMI and blood pressure later in life whereas conflicting results concerning metabolic disturbances in adulthood have been published (Sovio *et al.*, 2014; Aris *et al.*, 2019). Adiposity rebound (AR) is defined as the second rise of BMI following the nadir phase in the childhood growth trajectory. The mean age at AR is population-dependent and normally occurs at age 5–7 years (Koyama *et al.*, 2014; Péneau *et al.*, 2016). Early AR is defined by its occurrence before the age of 5 years (Péneau *et al.*, 2016) and has been associated with obesity and an unfavorable metabolic profile in adulthood (Koyama *et al.*, 2014; Péneau *et al.*, 2016). In our previous publication in this same population, early AR (under 5.1 years) was also associated with the development of PCOS, independently of adulthood BMI (Koivuaho *et al.*, 2019). However, we are not aware of any studies focusing on the association of the timing and BMI of AR or AP with fertility outcomes.

The aim of this study was to evaluate the relation of BMI from birth until adolescence (age 15 years) with fertility and reproductive capacity until the end of the reproductive time window (defined here as 50 years). More specifically, we focused on assessing the association between childhood obesity in three childhood age groups, namely early childhood (3–6 years), prepubertal (7–10 years) and pubertal periods (11–15 years), with adulthood fertility. Reproductive capacity was evaluated by decreased fecundability and the need for infertility assessment and treatment by age 46 years, as well as childlessness and number of children by age 50 years.

## Materials and methods

### Study population

**Figure 1.**
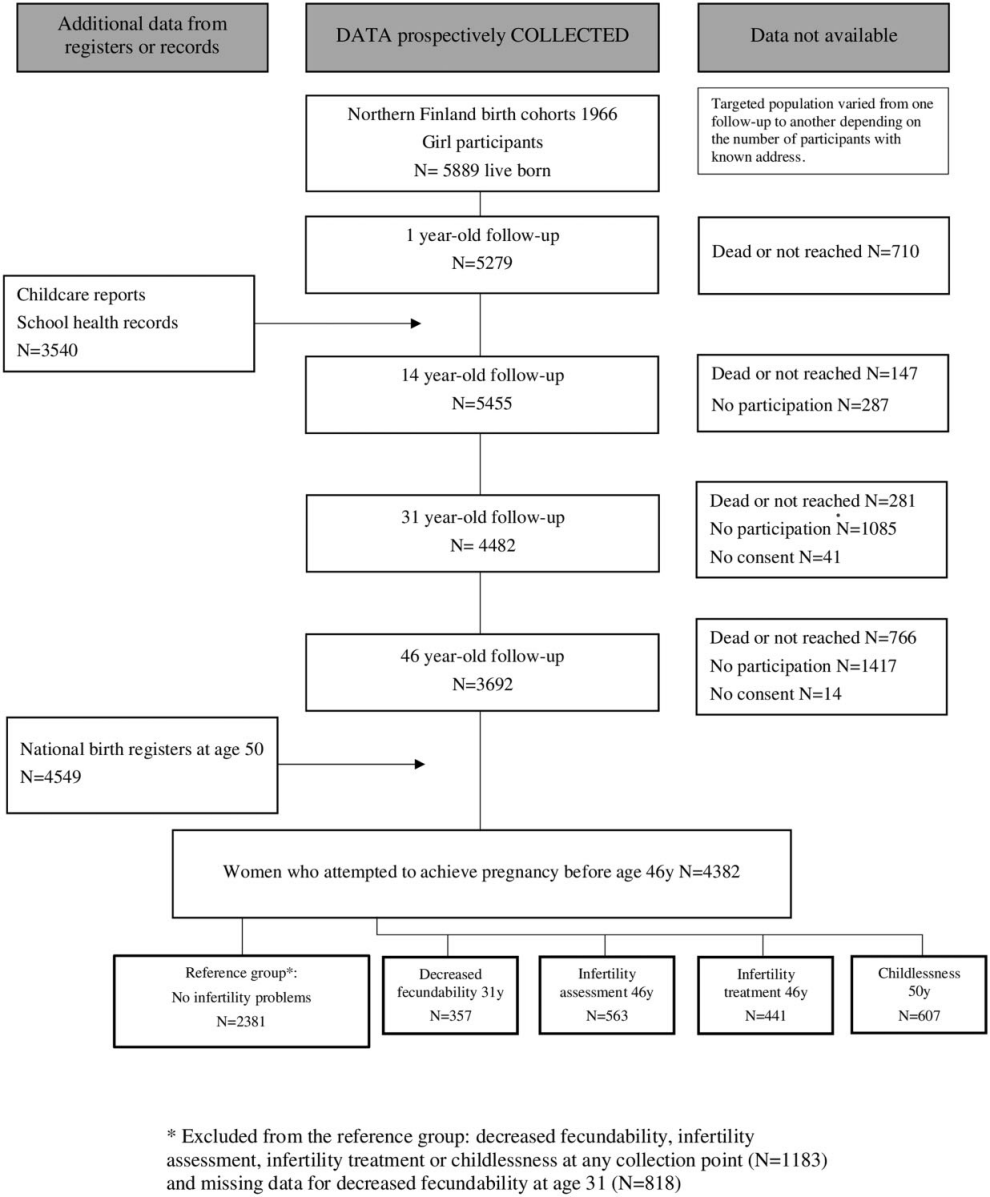
**Study population in the Northern Finland Birth Cohort 1966**.

The study population was drawn from the prospective, longitudinal, population-based, Northern Finland Birth Cohort 1966 (NFBC1966), recruited at gestational week 24 from the two northernmost provinces of Finland. The study included a total of 12 058 live births (5889 females), covering 96% of all births in this area (Rantakallio, 1988). The study was approved by the Ethics Committee of the Northern Ostrobothnia Hospital District. All participants provided informed consents. The study population is presented in Fig. 1.

A postal questionnaire was sent at age 1 year to 5800 girls’ child health and welfare nurses (of these 5279 i.e. 91.0% answered), at age 14 years to 5742 girls and their families (of these 5455 i.e. 95% answered), at age 31 years to 5 608 women (of these, 4523 i.e. 81% answered, of these 4482 consented to data use) and at age 46 years to 5123 women (of these 3706 i.e. 72% answered, of these 3692 consented to data use). The questionnaires at ages 31 and 46 years included multiple questions on lifestyle, education, family history, living environment, health, and fertility (Table I).

**Table I:** Questions and the derived parameters used in the analyses.

Age (years)	Questions	Parameters	**Study population N = 4382** ^*^			
**14**	“ Your weight and your height?”	**BMI at age 14**	N = 3941		Missing N = 441	
**31**	“How old were you when you started menstruating?”	**Menstrual age**	N = 4056 Missing N = 326			
	**“**How many months did it took you to get pregnant (time without contraception)?”	Fecundability under 12 months—**Normal** over 12 months—**Decreased**	N = 2654 N = 357 Missing N = 1371			
	“Your educational status? Mark the highest.”	**Education** Basic education Secondary education Tertiary education	N = 369 N = 2929 N = 794 Missing N = 290			
	Have you ever smoked in your life? Have you ever smoked regularly? Do you smoke nowadays?	**Smoking** Never smoked Former/occasional smoker Active smoker	N = 2031 N = 1047 N = 997 Missing N = 307			
	Is your menstrual cycle often (more than twice a year) over 35 days? Do you have excessive growth of body hair?	**PCOS _symptoms_** Oligomenorrhea Hirsutism If both, defined as PCOS _symptoms_	N = 307 N = 288 N = 110		**PCOS _symptoms_** + **PCOS _diagnosed_** = **PCOS _total_** N = 259	
**46**	Have you ever been diagnosed with polycystic ovaries and/or PCOS?	**PCOS _diagnosed_**	N = 174			
**31 and 46**			At age 31 y	At age 46 y		**Before 46 y**
	“Have you been assessed for infertility?”	**No infertility assessment** **Infertility assessment**	N = 3172 N = 393 Missing N = 817	N = 2925 N = 439 Missing N = 1018		N = 3819 N = 563
	”Have you been treated for infertility?”	**No infertility treatment** **Infertility treatment**	N = 3353 N = 288 Missing N = 741	N = 3074 N = 307 Missing N = 1001		N = 3941 N = 441
	“Your marital status: married, cohabiting, single, divorced, widowing?”	**Marital status** Ever been in relationship Single	N = 3488 N = 579 Missing N = 315	N = 3200 N = 290 Missing N = 892		N = 3950 N = 402 Missing N = 30

PCOS: polycystic ovary syndrome.

* Women who never attempted to achieve pregnancy were excluded.

The outcomes concerning fertility were defined based on the answers to the 31 year and 46 year questionnaires (Table I). The time to pregnancy in months was asked only at age 31 years and calculated from the beginning of unprotected intercourse until the first pregnancy. Decreased fecundability was defined as active exposure to pregnancy (i.e. not using contraception) for 1 year without getting pregnant (Koivunen *et al.*, 2008). Other outcomes were as follows: infertility assessment, infertility treatment, childlessness and number of children. A reference group was defined as women without decreased fecundability, infertility assessment, infertility treatment and childlessness. Women with missing data regarding decreased fecundability were also excluded from the reference group (Fig. 1). The final study population included 4382 women who attempted to achieve pregnancy before age 46 years.

The number of deliveries until the end of 2016 (when the women turned 50 years) was obtained from the Finnish Medical Birth Register founded in 1987. The birth data before 1987 were collected from the Population Register Centre live births to make the birth data complete.

### Growth data and definition of obesity in the study population

Height and weight were measured by professional nurses at ages 6 (n = 2928) and 12 months (n = 3797) and BMI (kg/m^2^) was calculated.

Height and weight from infancy to adolescence were collected from records, and were measured by the child health and welfare nurses, and later by school nurses, as a part of the national child health program, which is free for all children in Finland. Given that measured weight and height were not available for every year in all participants, three different age groups were created: early childhood (3.00–6.99 years, n = 2 511), prepubertal period (7.00–10.99 years, n = 2652) and pubertal period (11.00–15.99 years, n = 2 751), according to the literature regarding childhood growth (Tanner and Whitehouse, 1976). If participants had more than one measurement per year, the mean BMI was calculated. For each age group, the participants were stratified into underweight (below 5th percentile (pc)), normal weight (5th–85th pc), overweight (85th–95th pc) and obese (over 95th pc) groups according to the criteria of the World Health Organization (WHO) and Center for Disease Control and Prevention (Kuczmarski *et al.*, 2000). Obesity and underweight in a specific age group were defined as at least one BMI value above the 95th pc or below the 5th pc during the related period, respectively.

BMI and the timing of AP and AR were derived from fitted growth curves, as described previously (Sovio *et al.*, 2014). The natural changes observed in childhood BMI required the data to be split into two age windows: infancy (2 weeks–1.5 years) and childhood (1.5–13 years) to model the BMI trajectories (Sovio *et al.*, 2014). There were, on average, seven measurements during infancy and 16 during childhood for each child. Calculation of AP (1 month–1.5 years) and AR (1.5–13 years) was carried out only for the children having at least three measurements during childhood (AP n = 2083, AR n = 2566). We used a cut-off value of 5.1 years to define an early AR, as detailed by Koivuaho *et al.* (2019).

Height and weight at age 14 years were all reported by the parents. BMI at age 14 years was used only for continuous analyses. Height and weight at age 31 years were measured at the clinical examination. If measurements were not available, self-reported data were used. According to our previous study in this same population, there was no statistically significant difference between self-reported and clinically measured BMIs (Ollila *et al.*, 2016).

### Covariates

All results were adjusted for marital status at ages 31 and 46 years, and smoking and education level at age 31 years (Table II). Prenatal factors (maternal age, smoking at the end of pregnancy, prepregnancy BMI, own birthweight or gestational age), which are known to associate with childhood growth (Woo Baidal *et al.*, 2016) and childlessness (Ekholm *et al.*, 2005; DeKeyser *et al.*, 2012), were not significantly associated with any fertility outcomes in our study population (Table II) and were, therefore, not included as covariates. Marital status was defined as either ‘being single’ (both at ages 31 and 46 years) or ‘ever being in relationship’ (ever been married/cohabited/divorced/widow). Smoking and education level at age 31 years were classified based on the questionnaire (Table I). The criteria for defining PCOS in this population have been described previously (Ollila *et al.*, 2016; Karjula *et al.*, 2017; Koivuaho *et al.*, 2019). Briefly, women presenting both self-reported oligo/amenorrhea and hirsutism at age 31 years (n = 110) and/or with self-reported formally diagnosed PCOS by age 46 years (n = 174) were defined as women with PCOS (n = 259) (Table I).

**Table II:** Characteristics of the study population.

	No infertility problems before age 50 years	Decreased fecundability at age 31 years	Infertility assessment before age 46 years	Infertility treatments before age 46 years	Childlessness at age 50 years
	N = 2381	N = 357	N = 563	N = 441	N = 607
**Maternal parameters**					
Mother’s prepregnancy BMI (kg/m^2^), mean ± SD	23.03 ± 3.11	22.91 ± 3.13	22.98 ± 3.29	22.91 ± 3.24	23.20 ± 3.22
Mother’s age (year), mean ± SD	27.71 ± 6.59	27.62 ± 6.65	27.55 ± 6.58	27.26 ± 6.42	28.13 ± 6.85
Mother’s smoking end of pregnancy (%)	15.5	17.4	16.3	17.2	15.0
**Index person’s parameters**					
**Childhood**					
Birthweight (g), mean ± SD	3396 ± 496	3380 ± 496	3394 ± 507	3409 ± 496	3399 ± 522
Gestational age (week), mean ± SD	38.61 ± 7.88	38.73 ± 7.16	38.57 ± 7.83	38.63 ± 7.74	38.65 ± 7.57
BMI 14 y (kg/m^2^), mean ± SD	19.38 ± 2.31	19.78 ± 2.74^*^	19.52 ± 2.58	19.57 ± 2.62	19.65 ± 2.62^*^
Age at menarche (year), mean ± SD	12.91 ± 1.29	12.81 ± 1.29	12.98 ± 1.38	12.98 ± 1.39	12.84 ± 1.31
**Adulthood**					
Ever been in relationship (%)	94.4	96.1	96.3^*^	97.3^*^	66.7^*^
PCOS (%)	4.7	16.1^*^	16.8^*^	18.1^*^	6.8
BMI 31 y (kg/m^2^), mean ± SD	23.87 ± 4.18	24.56 ± 4.87^*^	24.17 ± 4.81	24.15 ± 4.77	23.98 ± 4.99
Smoking 31 y (%)					
Non-smoker (reference)	48.5	53.1	51.5	52.2	49.0
Former/occasional smoker	27.2	23.4	24.6	25.9	22.4^*^
Active smoker	24.8	23.4	23.9	21.9	28.6^*^
Education 31 y (%)					
Basic	9.1	7.3	7.3	7.5	7.5
Secondary (reference)	73.9	75.1	69.7^*^	70.1^*^	67.7^*^
Tertiary	17.0	17.6	23.0^*^	22.4^*^	24.8^*^
Number of children, mean ± SD	2.70 ± 1.66	1.91 ± 1.60^*^	1.63 ± 1.46^*^	1.62 ± 1.37^*^	0

* *P* < 0.05 (women without fertility problems is used as reference population).

Significance tests for continuous variables were performed by using the independent samples *t*-test or the Mann–Whitney *U* test, as appropriate, and Pearson’s test was used for categorical variables.

### Statistical methods

The differences between groups for continuous variables (age and BMI at AP and AR, menarche age, BMI at age 14 and 31 years, and number of children,) were analyzed by using an independent Student’s *t*-test, the Mann–Whitney *U*, one-way ANOVA or the Kruskal–Wallis tests, as appropriate. For the differences in categorical parameters, a Chi-squared test was used. For these tests, the results are reported as means or medians and SD or 95% Cl, or prevalence (%) and odds ratios (OR) with 95% CI, respectively. *P* value <0.05 was considered statistically significant. Multivariate analyses were conducted using a binary logistic regression modelling. The results were reported as ORs with 95% CIs.

The data were adjusted for the following variables: marital status during the reproductive period (at ages 31 and 46 years) and smoking and educational level at age 31 years. Given that PCOS is strongly associated with overweight and obesity and is also the most frequent cause of anovulatory infertility in women (Brewer and Balen, 2010), we performed additional analyses after excluding women with PCOS. Linear, cubic and quadratic associations between number of children and BMI at age 14 years were assessed with curve estimation. IBM SPSS Statistics for Windows, Version 25.0. Armonk, NY, USA: IBM Corp. was used to assess differences between the study groups and to perform the regression analyses.

## Results

### Characteristics of the study population

The numbers of participants varied between each age groups and different fertility outcomes, owing to variations in the number of answers to each question and/or participation in the clinical examination (Table II). In all, 1104 women reported some fertility problem (decreased fecundability, infertility assessment or infertility treatment) or remained childless. Owing to a significant overlap between fertility outcomes, most of the women were included in several infertility outcome groups. The overlap between infertility assessment and treatment was particularly extensive (96% of the women receiving treatment were assessed for infertility and 72% of the women assessed received treatment). On the other hand, only 18% of women who remained childless had been treated for infertility, and only 23% of those who received treatment, remained childless. The final study population included 4382 women who attempted to achieve pregnancy before age 46 years: decreased fecundability (n = 357), infertility assessment (n = 563), infertility treatment (n = 441) and childlessness (n = 607), and the reference group including the women without any infertility problem (n = 2381) (Fig. 1).

As expected, parous women were more likely to have ever been in a relationship compared with their counterparts without deliveries. During the follow-up, the rate of active smoking reduced from 24.8% to 17.2% and the education level increased (tertiary: from 24.8% to 29.0%) (data not shown). Of note, because of the definitions used (see the ‘Study population’ subsection of the ‘Materials and Methods’), underweight (BMI < 95th pc) was underrepresented, and obesity (BMI > 95th pc) was overrepresented, especially in the early years (6 months—3 years) (Supplementary Table SI).

### Infancy, age 6–12 months

BMI under 5th pc or over 95th pc at age 6 or 12 months (Supplementary Table SII) did not correlate with any fertility outcomes.

### AP and AR

The age at AP was not associated with any adverse fertility outcomes or childlessness, but BMI at the age of AP was inversely associated with childlessness (17.78 vs 17.67; OR 0.84, (95% CI: 0.71–0.99)) in the crude analyses (Table III). However, after adjusting for all confounding factors and excluding women with PCOS, the difference lost its significance (OR: 0.83 (0.69–1.01)) (Supplementary Table SIII).

**Table III:** Association between age and BMI at adiposity peak and adiposity rebound with fertility outcomes.

		No infertility problems	Decreased fecundability at age 31 years	Infertility assessments before age 46 years	Infertility treatments before age 46 years	Childlessness at age 50 years
**Adiposity peak**	**Total number**	**n = 1281**	**n = 181**	**n = 286**	**n = 225**	**n = 314**
Age (years)	Mean ± SD	9.12 ± 0.43	9.10 ± 0.37	9.10 ± 0.35	9.08 ± 0.37	9.11 ± 0.39
	OR 95% Cl Crude	ref	0.82 (0.55–1.23)	0.82 (0.58–1.15)	0.76 (0.52–1.12)	0.92 (0.67–1.27)
	Model I	ref	0.83 (0.55–1.24)	0.82 (0.58–1.15)	0.76 (0.52–1.12)	0.91 (0.64–1.28)
	Model II	ref	0.82 (0.55–1.23)	0.81 (0.58–1.14)	0.76 (0.52–1.11)	0.91 (0.64–1.28)
BMI (kg/m^2^)	Mean ± SD	**17.78 ± 0.78**	17.79 ± 0.88	17.71 ± 0.85	17.68 ± 0.86	**17.67 ± 0.81**
	OR 95% Cl Crude	ref	1.02 (0.83–1.25)	0.87 (0.73–1.03)	0.84 (0.69–1.02)	**0.84 (0.71–0.99)**
	Model I	ref	1.02 (0.84–1.25)	0.86 (0.73–1.03)	0.84 (0.70–1.02)	0.84 (0.70–1.00)
	Model II	ref	1.02 (0.84–1.25)	0.87 (0.73–1.03)	0.84 (0.70–1.02)	0.84 (0.69–1.00)
**Adiposity rebound**	**Total number**	**n = 1520**	**n = 211**	**n = 346**	**n = 275**	**n = 360**
Age (years)	Mean ± SD	**5.57 ± 0.81**	**5.41 ± 0.95**	5.54 ± 0.94	5.55 ± 0.95	**5.48 ± 0.96**
	OR 95% Cl Crude	ref	**0.82 (0.69–0.96)**	0.96 (0.84–1.10)	0.97 (0.82-1.12)	**0.86 (0.75–0.99)**
	Model I	ref	**0.82 (0.70–0.96)**	0.96 (0.84–1.10)	0.97 (0.83–1.13)	**0.83 (0.72–0.96)**
	Model II	ref	**0.82 (0.70-0.97)**	0.97 (0.84–1.11)	0.98 (0.84-1.14)	**0.84 (0.73–0.97)**
BMI (kg/m^2^)	Mean ± SD	15.31 ± 1.05	15.45 ± 1.26	15.27 ± 1.19	15.25 ± 1.14	15.33 ± 1.12
	OR 95% Cl Crude	ref	1.11 (0.98–1.26)	0.95 (0.85–1.06)	0.93 (0.82–1.05)	1.04 (0.94–1.17)
	Model I	ref	1.11 (0.98–1.27)	0.95 (0.85–1.06)	0.93 (0.82–1.05)	1.02 (0.90–1.15)
	Model II	ref	1.11 (0.97–1.26)	0.94 (0.84–1.06)	0.92 (0.81–1.05)	1.01 (0.90–1.15)

Women who reported to have never attempted to achieve pregnancy were excluded from the analyses.

Results are shown as odd ratios (OR) with 95% CI.

Model I: Adjustment for marital status during reproductive period.

Model II: Model I + adjustment for education and smoking at age 31 years.

The bold values significance is expressed in the Tables as ORs.

The age at AR was earlier in women with decreased fecundability and childlessness, but after the exclusion of the women with PCOS, only the association with childlessness remained significant (Supplementary Table SIII). After excluding women with PCOS, a higher BMI at AR was associated with lower risk for infertility assessment (adjusted OR 0.87 (0.76–0.98)) and a trend for lower risk for infertility treatments (0.88 (0.75–1.01)) (Supplementary Table SIII).

Girls with AR <5.1 years had a higher risk of remaining childless compared to girls with AR over 5.1 years (OR: 1.38 (1.07–1.79)) and the difference remained significant after adjusting for all confounding factors and after the exclusion of women with PCOS (adjusted OR: 1.45 (1.10–1.92)). There were no significant association with other fertility outcomes (data not shown).

### Early childhood, age 3–6 years

**Figure 2.**
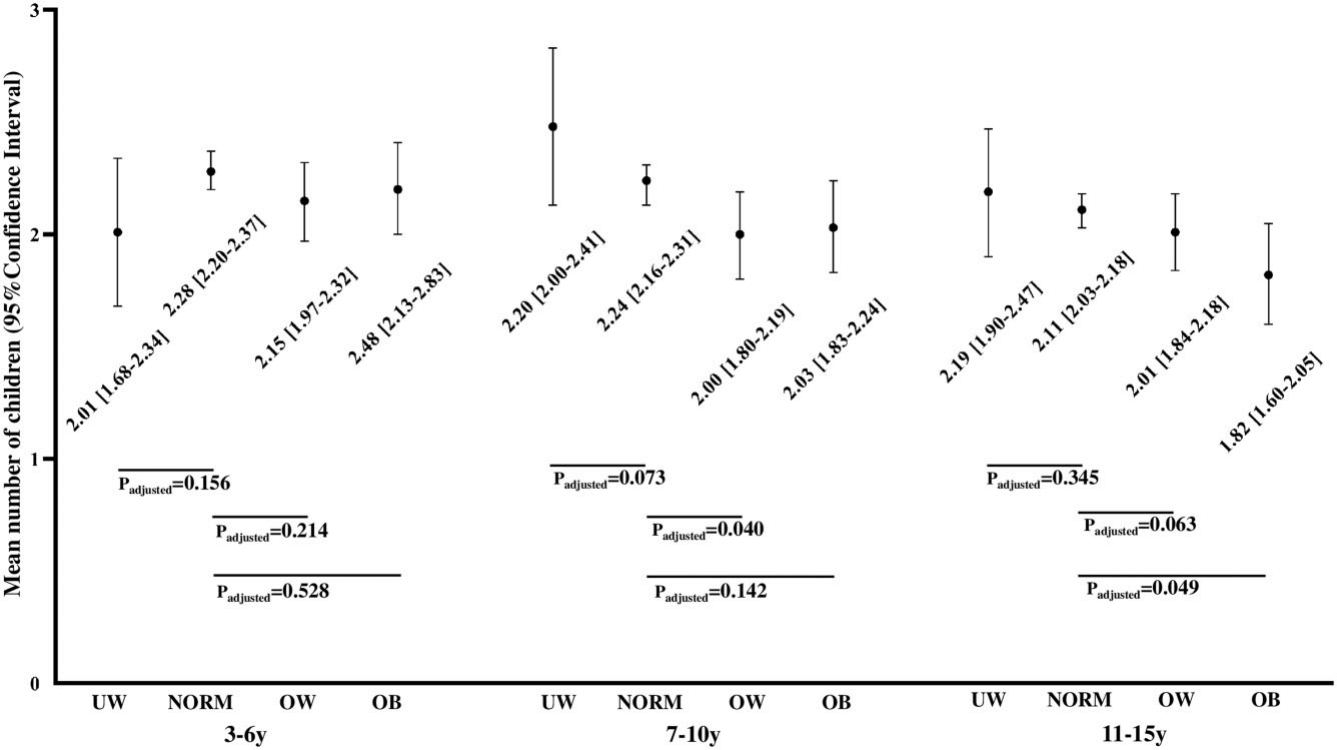
**Total number of children by age 50 years in different weight groups at ages 3–6 years, 7–10 years and 11–15 years**. The results were adjusted for: marital status during reproductive age, education and smoking at age 31 years. Women who reported to have never attempted to achieve pregnancy were excluded from the analyses. Normal weight is used as a reference group. y, year; UW, underweight (BMI <5th percentile (pc)); NORM, normal weight (BMI 5th–85th pc); OW, overweight (BMI 85th–85th pc); OB, obese (BMI >95th pc).

At age 3–6 years, 9.4% of the girls were classified as obese and of those 36.9% were obese at age 31 years. Interestingly, the prevalence of obesity was the highest at age 3 years and decreased over time (Supplementary Table SI). Of the women who had participated in the questionnaires and the clinical examinations at ages 31 and 46 years, 61.8% and 66.4% had growth data available, respectively. This age group showed no significant associations between underweight, overweight or obesity and any fertility outcomes (Table IV) or the number of children (Fig. 2).

**Table IV:** Association between childhood underweight, overweight and obesity in the different age groups with fertility outcomes compared to childhood normal weight.

		No infertility problems (n = 2381)	Decreased fecundability at age 31 years (n = 357)	Infertility assessments before age 46 years (n = 563)	Infertility treatments before age 46 years (n = 441)	Childlessness at age 50 years (n = 607)
**Age 3–6 years**	**Total number**	**N = 1385**	**N = 193**	**N = 319**	**N = 250**	**N = 332**
**Underweight**	Prevalence (%)	3.5	4.1	5.3	5.2	4.8
	OR 95% Cl Crude	ref	1.04 (0.49**–**2.22)	1.28 (0.73**–**2.26)	1.21 (0.64**–**2.28)	1.31 (0.62**–**2.77)
	Model I	ref	1.03 (0.48**–**2.19)	1.27 (0.72**–**2.25)	1.20 (0.63**–**2.27)	1.42 (0.77**–**2.64)
	Model II	ref	1.05 (0.49**–**2.45)	1.29 (0.73**–**2.28)	1.22 (0.46**–**2.31)	1.45 (0.78**–**2.70)
**Overweight**	Prevalence (%)	13.6	11.4	12.2	12.0	14.8
	OR 95% Cl Crude	ref	0.92 (0.61**–**1.37)	0.82 (0.58**–**1.15)	0.76 (0.52**–**1.11)	0.93 (0.67**–**1.29)
	Model I	ref	0.91 (0.61**–**1.40)	0.82 (0.58**–**1.15)	0.75 (0.51**–**1.11)	0.94 (0.66**–**1.35)
	Model II	ref	0.91 (0.61**–**1.37)	0.81 (0.58**–**1.15)	0.76 (0.52**–**1.11)	0.94 (0.66**–**1.35)
**Obese**	Prevalence (%)	10.1	11.4	6.3	6.4	7.8
	OR 95% Cl Crude	ref	1.30 (0.80**–**2.11)	0.68 (0.41**–**1.11)	0.67 (0.39**–**1.17)	0.89 (0.56**–**1.40)
	Model I	ref	1.29 (0.80**–**2.10)	0.67 (0.41**–**1.11)	0.67 (0.38**–**1.16)	0.80 (0.49**–**1.32)
	Model II	ref	1.29 (0.80**–**2.10)	0.66 (0.40**–**1.09)	0.66 (0.37**–**1.14)	0.79 (0.48**–**1.30)
**Age 7–10 y ears**	**Total number**	**N = 1457**	**N = 205**	**N = 336**	**N = 270**	**N = 348**
**Underweight**	Prevalence (%)	7.1	5.9	9.2	8.9	6.6
	OR 95% Cl Crude	ref	0.87 (0.47**–**1.61)	1.29 (0.83**–**1.98)	1.26 (0.78**–**2.03)	0.95 (0.58**–**1.55)
	Model I	ref	0.87 (0.47**–**1.61)	1.28 (0.83**–**1.97)	1.26 (0.79**–**2.02)	0.93 (0.55**–**1.56)
	Model II	ref	0.86 (0.46**–**1.60)	1.32 (0.85**–**2.04)	1.29 (0.80**–**2.09)	0.92 (0.54**–**1.56)
**Overweight**	Prevalence (%)	10.3	10.7	11.6	11.5	11.8
	OR 95% Cl Crude	ref	1.23 (0.81**–**1.89)	1.13 (0.79**–**1.61)	1.10 (0.75**–**1.63)	1.23 (0.87**–**1.76)
	Model I	ref	1.23 (0.80**–**1.88)	1.12 (0.78**–**1.59)	1.09 (0.74**–**1.61)	1.32 (0.91**–**1.93)
	Model II	ref	1.23 (0.80**–**1.88)	1.10 (0.77**–**1.58)	1.09 (0.73**–**1.61)	1.28 (0.88**–**1.87)
**Obese**	Prevalence (%)	6.0	11.2	5.7	5.6	7.5
	OR 95% Cl Crude	ref	**2.06 (1.26–3.34)**	0.95 (0.56**–**1.61)	0.92 (0.51**–**1.65)	1.34 (0.84**–**2.15)
	Model I	ref	**2.04 (1.25–3.33)**	0.95 (0.56**–**1.61)	0.91 (0.51**–**1.63)	1.16 (0.69**–**1.95)
	Model II	ref	**2.05 (1.26–3.35)**	0.94 (0.55**–**1.59)	0.91 (0.51**–**1.63)	1.15 (0.68**–**1.94)
**Age 11–15 years**	**Total number**	**N = 1525**	**N = 213**	**N = 345**	**N = 274**	**N = 355**
**Underweight**	Prevalence (%)	10.0	9.4	12.2	13.1	10.7
	OR 95% Cl Crude	ref	0.98 (0.60**–**1.61)	1.35 (0.93**–**1.94)	1.41 (0.95**–**2.09)	1.09 (0.73**–**1.63)
	Model I	ref	0.94 (0.60**–**1.61)	1.35 (0.93**–**1.94)	1.42 (0.95**–**2.10)	1.21 (0.79**–**1.84)
	Model II	ref	0.97 (0.60**–**1.59)	1.37 (0.95**–**1.99)	1.44 (0.97**–**2.15)	1.22 (0.80**–**1.86)
**Overweight**	Prevalence (%)	10.3	9.9	7.8	9.5	12.4
	OR 95% Cl Crude	ref	1.11 (0.73**–**1.68)	0.92 (0.63**–**1.33)	1.02 (0.69**–**1.51)	1.38 (0.99**–**1.94)
	Model I	ref	1.10 (0.73**–**1.67)	0.91 (0.63**–**1.32)	1.00 (0.68**–**1.48)	**1.55 (1.08–2.22)**
	Model II	ref	1.11 (0.73**–**1.69)	0.91 (0.63**–**1.31)	1.01 (0.68**–**1.50)	**1.53 (1.07–2.20)**
**Obese**	Prevalence (%)	4.9	9.4	7.0	6.6	**7.3**
	OR 95% Cl Crude	ref	**2.01 (1.20–3.39)**	1.56 (0.96**–**2.51)	1.47 (0.86**–**2.51)	**1.84 (1.51–2.93)**
	Model I	ref	**2.00 (1.19–3.37)**	1.56 (0.96**–**2.51)	1.45 (0.85**–**2.28)	**1.97 (1.19–3.24)**
	Model II	ref	**2.04 (1.21–3.44)**	1.53 (0.95**–**2.48)	1.46 (0.85**–**2.50)	**1.97 (1.19–3.27)**

Women who reported to have never attempted to achieve pregnancy were excluded from the analyses.

Underweight (BMI <5th percentile (pc)), normal weight (BMI 5th–85th pc), overweight (BMI 85th–85th pc), obese (BMI >95th pc).

Results are OR with 95% CI.

Model I: Adjustment for marital status during reproductive period.

Model II: Model I + adjustment for education and smoking at age 31 years.

The bold values significance is expressed in the Tables as ORs.

### Prepubertal period, age 7–10 years

At age 7–10 years, 6.7% of the girls were obese and, of those, 56.8% remained obese at age 31 years. Obesity at age 7–10 years was associated with decreased fecundability at age 31 years, but not with childlessness and self-reported infertility assessment or treatment (Table IV). The results did not change after adjusting for all confounding factors (Table IV) and excluding women with PCOS (Supplementary Table SIII). Overweight at age 7–10 years was associated with a lower number of children compared to normal weight but the association between obesity and number of children did not reach statistical significance (Fig. 2). Conversely, women who were underweight at age 7–10 years tended to have more children than their normal weight counterparts (Fig. 2).

### Menarche age

The mean age at menarche was 12.9 years and the median was 13 years (range: 9–18) in the entire study population. Girls with obesity in each age group had earlier age at menarche than normal weight girls: age 3–6 years: 12.4 (95% Cl: 12.1–12.6) versus 12.9 (12.8–12.9) years, p=0.001; age 7–9 years: 12.0 (11.8–12.3) versus 12.9 (12.8–13.0) years, p=0.0001; age 11–15 years: 12.1 (11.9–12.4) versus 12.9 (12.8–12.9) years, p=0.0001.

Being underweight or overweight at age 3–6 years was not associated with age at menarche (data not shown) but at ages 7–10 and 11–15 years underweight girls experienced later (age 7–10 years: 13.3 (13.0–13.5), p=0.015; age 11–15 years: 13.6 (13.4–13.8), p=0.0001) and overweight girls earlier (age 7–10 years: 12.3 (12.1–12.6), p=0.0001; age 11–15 years: 12.1 (11.9–12.3), p=0.0001) age at menarche. We observed no statistical evidence supporting that age at menarche was associated with any of the measured outcomes (Table II).

### Pubertal period, age 11–15 years

At age 11–15 years, 5.6% of the girls were obese and of those 74.6% were obese at age 31 years. Obesity at age 11–15 years was associated with a higher risk of decreased fecundability and childlessness compared to normal weight (Table IV). Also, overweight was associated with childlessness after adjustments (Table IV), but it was not associated with infertility assessment or treatment. Girls with obesity at age 11–15 years had fewer children than normal weight girls with a trend in the group of overweight girls (Fig. 2). After excluding women with PCOS, the results did not change (Supplementary Table SIII).

Underweight at puberty tended to be associated with a higher risk of infertility assessment and treatment. After excluding women with PCOS, the association with infertility treatment became significant (adjusted OR: 1.55 (1.02–2.36)) but the association with childlessness did not (Supplementary Table SIII).

**Figure 3.**
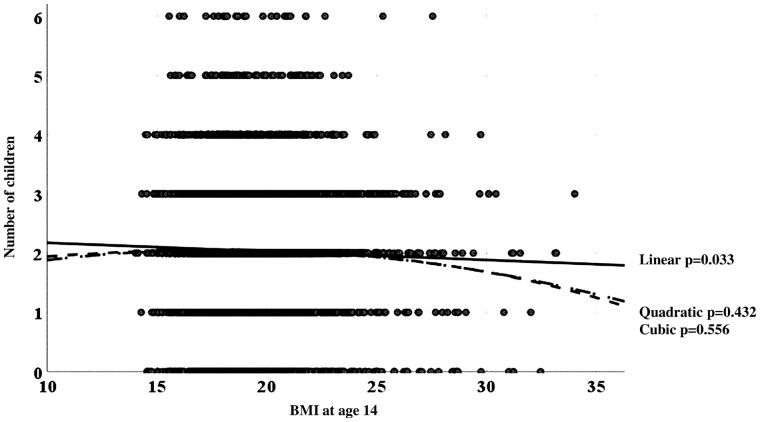
**Curve estimation between BMI at age 14 years and number of children.** *P*-value for each curve fitting is presented.

To clarify the type of association between BMI in adolescence and the number of children, we performed curve estimation analyses exploring linear, cubic and quadratic associations between BMI at age 14 years as a continuous variable and the number of children. We found a significant linear association (but no cubic or quadratic association) between these two parameters: the number of children decreased linearly along with an increase in BMI at that age (number of children (y) = 2.88–0.03 × BMI) (Fig. 3).

## Discussion

The present study suggests that being obese in mid-childhood and at adolescence is associated with an increased risk of fertility problems in adulthood. Obesity at age 7–10 years was associated with a decreased fecundability at age 31 years but not with a higher risk of other fertility problems or childlessness. Moreover, overweight girls in the 7–10 years age group had fewer children than their normal weight counterparts. The women who were obese at age 11–15 years were more likely to remain childless, to have fewer children and to suffer more often from decreased fecundability compared to their normal weight counterparts. Underweight at puberty, but not at other ages, was associated with a trend for more infertility assessment and treatment but not with decreased fecundability, childlessness or a lower number of children.

Several studies have suggested that children with obesity remain obese in adulthood (Serdula *et al.*, 1993; Simmonds *et al.*, 2015). This is supported by the present data as most of the children who were obese in mid-childhood and especially in adolescence were also obese as adults.

Early timing of AR has been previously linked to adverse metabolic outcomes in childhood and in adulthood (Hughes *et al.*, 2014; Koyama *et al.*, 2014; Péneau *et al.*, 2016) as well as with an increased risk for PCOS (Koivuaho *et al.*, 2019). In this study, an earlier age of AR remained significantly associated with childlessness after excluding women with PCOS, supporting an independent association between the timing of the AR and fertility capacity in adulthood, partly independently of the syndrome.

A later timing and a higher BMI at the time of AP have been associated with a higher BMI in childhood and higher adiposity in adulthood (Sovio *et al.*, 2014; Aris *et al.*, 2019), but we are not aware of any studies on their association with fertility in adulthood. In the present study, the timing of AP was not associated with any fertility outcomes but, in contrast to previous data (Lake *et al.*, 1997; Jacobs *et al.*, 2017), a lower BMI at an early age, especially at the age of AP, was associated with childlessness. Similarly, a lower BMI at the age of AP was associated with an increased risk of infertility assessment and a tendency towards infertility treatment later in life, but that was seen only after excluding the women with PCOS. The hypothalamic-pituitary-ovarian axis in infancy and in early childhood is transiently activated. This so called ‘minipuberty’ is characterized by elevated gonadotrophin and oestradiol levels (Forest *et al.*, 1973; Kuiri-Hänninen *et al.*, 2014). Soon after birth, the levels of LH and FSH start to increase, achieving their peak between 1 week and 3 months and thereafter decreasing gradually by the age of 3–4 years (Kuiri-Hänninen *et al.*, 2014). Hormonal changes and gonadal development at minipuberty have been shown to predict reproductive disorders later in life (Kiviranta *et al.*, 2016; Lanciotti *et al.*, 2018). We might formulate the hypothesis that a lower BMI in infancy may be associated with suboptimal mini-puberty, further mediating the association with decreased reproductive capacity in adulthood. However, further studies are warranted to test the hypothesis of the possible underlying mechanisms.

Early age of puberty has been linked to both obesity and an adverse metabolic profile in adulthood (Lee *et al.*, 2007; Bleil *et al.*, 2012) but the results concerning fertility are conflicting (Pinola *et al.*, 2012; Weghofer *et al.*, 2013; Chen *et al.*, 2015). The present study supports the commonly accepted notion that girls with obesity experience earlier menarche and those who are underweight later menarche. Indeed, menarche at an early age has been previously shown to be linked with adulthood obesity and a worse metabolic profile (Adair and Gordon-Larsen, 2001; Bleil *et al.*, 2012) as well as with menstrual disorders and PCOS (Carroll *et al.*, 2012). In the present data set, the age at menarche was not significantly associated with parity or fertility outcomes. This is in line with the findings of a Danish cohort study following 2653 women trying to conceive (Wise *et al.*, 2011) and a Finnish follow-up cohort study that found no significant association between the age at menarche and fertility at age 26 years (Pinola *et al.*, 2012). Conversely, a Chinese cross-sectional study reported a significant correlation between later menarche and the risk of infertility in adulthood (Chen *et al.*, 2015) whereas a Finnish study found that both early and late menarche were associated with childlessness (Jokela *et al.*, 2007). All in all, owing to multiple interrelationships with ethnicity, weight, chronic illnesses and susceptibility to eating disorders, the timing of menarche seems to be too complex a parameter to predict fertility (Euling *et al.*, 2008).

Interestingly, during the age window of 11–15 years, obesity and overweight, but not underweight, were associated with childlessness even after excluding women with PCOS. Moreover, we observed a linear inverse correlation between BMI at age 14 years and the number of children. This is consistent with another Finnish cohort study following girls/women from adolescence (age 12–18 years) to adulthood (age 33–39 years) showing that obesity during adolescence was associated with reduced parity (Jokela *et al.*, 2007): it should be noted, however, that women who had never attempted to achieve pregnancy were not excluded from the analyses in that study. A recent study from Australia suggested that obesity, but not overweight, at age 7–11 years (but not at age 12–15 years), was associated with an increased risk of decreased fecundability and infertility assessments by age 34 years (He *et al.*, 2018). Moreover, in a study of 1061 women followed until age 45 years, those who were obese at age 9–12 and 13–18 years displayed more infertility problems and childlessness, but not more infertility diagnoses or treatments (Jacobs *et al.*, 2017).

A biological explanation for the above findings could be that adolescent girls with obesity have a more pronounced and longer state of physiological insulin resistance associated with puberty compared to normal weight girls. This is also associated with stronger prediction of adulthood obesity and impaired metabolic profile (Moran *et al.*, 1999; Maffeis *et al.*, 2002). Insulin resistance in adulthood associates also strongly with PCOS and anovulatory infertility (Palmert *et al.*, 2002). An increase in subcutaneous adiposity at any age in childhood has been suggested to be a more accurate predictor of later fecundity than BMI (Jacobs *et al.*, 2017). In the present study population, we could not verify this issue, but the association of obesity and overweight with childlessness remained significant after excluding women with PCOS. This suggests that the adverse effect of higher BMI at adolescence on later fertility is not solely caused by marital status or PCOS, in line with the findings of Jacobs *et al.* (2017).

Underweight girls, especially girls with anorexia nervosa or bulimia nervosa, have been shown to be at a higher risk of infertility assessment and treatment (Easter *et al.*, 2011; Tabler *et al.*, 2018) and having fewer or no children (Jokela *et al.*, 2007; Tabler *et al.*, 2018). However, opposite findings have also been reported (Bulik *et al.*, 1999). In the present study, underweight girls had an increased risk for later infertility treatment after exclusion of women with PCOS. However, they did not display any increased risk for decreased fecundability or for childlessness and even tended to have more children, in line with some other previous results (Bulik *et al.*, 1999; Easter *et al.*, 2011).

### Strengths and limitations

The strength of our study is the large population-based cohort followed-up from prenatal time to the end of the whole reproductive lifespan. This provides a rare opportunity to investigate the association between early growth and fertility outcomes later in life. The study population is also remarkably homogeneous concerning ethnicity. Data on deliveries are reliable owing to the use of the Finnish Medical Birth Register and Population Register Centre, which together cover 100% of births in Finland. All measurements, except BMI at age 14 years (used in curve estimation), were performed by trained professionals at all stages. We excluded all women who had never attempted to achieve pregnancy. We were also able to analyze childhood BMI growth trajectory data, which have shown their value as a tool to predict BMI and metabolic risks in adulthood (Hughes *et al.*, 2014; Koyama *et al.*, 2014; Péneau *et al.*, 2016).

This study has also some limitations. Despite the high participation rates throughout all collection points, children’s growth data were not available for all participants. As obesity was defined by the presence of at least one BMI value over the 95th percentile in an age group, it was probably over-diagnosed. According to the recent literature, BMI does not remain constant through lifetime, especially at the period from birth until the age of AR (Rolland-Cachera *et al.*, 2006; Koyama *et al.*, 2014; Couto Alves *et al.*, 2019). In our population, the prevalence of obesity between 6 months and 3 years was higher than expected. The period between AP and AR is a transitory phase between infancy and childhood and is characterized by quick changes in BMI as ‘baby fat’ is gradually lost. A higher BMI at some point during this period may be normal and transient (Rolland-Cachera *et al.*, 2006; Koyama *et al.*, 2014; Couto Alves *et al.*, 2019). In support of these findings, only 36.9% of the girls classified as ‘at least once obese at age 3–6 y’ were still obese in adulthood. This potential overdiagnosis of obesity may have reduced the significance of the association between childhood obesity and fertility outcomes.

Another potential limitation of our study is that the diagnosis of PCOS was self-reported, as both the documentation of PCOS symptoms at age 31 years and assessment of PCOS diagnosis at age 46 years were based on questionnaires. Moreover, hirsutism may have been over-reported owing to self-estimation. However, we have previously shown that the coexistence of self-reported oligo-/amenorrhea and hirsutism can accurately identify women with the typical endocrine, metabolic and psychological profiles of PCOS (Taponen *et al.*, 2003; Taponen *et al.*, 2004; Ollila *et al.*, 2016; Karjula *et al.*, 2017). Ovarian ultrasonography was not performed at age 31 years and it is possible that women with polycystic ovary morphology were included in the reference group, which may have resulted in an underestimation of the differences between the groups. Childhood obesity may also increase the risk of PCOS (Koivuaho *et al.*, 2019), which in turn increases the risk of infertility. However, it is still under debate whether obesity is a contributing factor to the development of PCOS in patients predisposed to the condition or whether obesity and PCOS are independent disorders. In this study, the risk of childlessness linked to obesity remained significant after excluding women with PCOS, suggesting an independent role of obesity. Moreover, the main objective of the present study was to investigate the association between childhood/adolescence obesity and later reproductive capacity, independently of the possible causes of obesity.

We were not able to adjust for BMI at the exact time of pregnancy as the data were available only at age 31 years when most of the women had already given birth. However, given that our results showed that obesity in childhood was strongly associated with obesity in adulthood and that pregnancy and parity have been shown to increase the risk of overweight and obesity (Williamson et al., 1994; Gunderson et al., 2004; Yakusheva *et al.*, 2017), adjusting our outcomes with BMI at age 31 years would have biased the results.

Finally, the infertility outcomes were self-reported, which can be considered as a limitation. Nonetheless, previous studies have indicated a good correlation between self-reported infertility treatments and medical records (Herbert *et al.*, 2012; Stern *et al.*, 2016) and our estimates are in line with previous data from developed countries showing an infertility prevalence of 5–15% in women (Boivin *et al.*, 2007; Herbert *et al.*, 2012).

## Conclusion

This study reveals that girls affected by obesity during mid-childhood and especially in adolescence are at a risk of lower fertility, a reduced number of children and an increased risk to remain childless in adulthood, partly independently of PCOS. Our findings also suggest that being underweight in adolescence is associated with an increased risk of later infertility treatment, but not with a higher risk of childlessness. All in all, these results support the importance of active prevention and treatment of obesity and of the importance of maintaining a normal weight, especially during adolescence, to sustain fertility.

## Data availability

Owing to the sensitive nature of the data collected for this study, requests to access the dataset from qualified researchers trained in human subject confidentiality protocols may be sent to NFBC project center at: NFBCprojectcenter@oulu.fi and researchers can visit the cohort website (www.oulu.fi/nfbc) for more information.

## Supplementary Material

deab164_Supplementary_Table_S1

deab164_Supplementary_Table_S2

deab164_Supplementary_Table_S3

## References

[deab164-B1] Adair LS , Gordon-Larsen P. Maturational timing and overweight prevalence in US adolescent girls. Am J Public Health 2001;91:642–644. 11291382 10.2105/ajph.91.4.642 PMC1446647

[deab164-B2] Aris IM , Rifas-Shiman SL, Li L, Kleinman KP, Coull BA, Gold DR, Hivert M, Kramer MS, Oken E. Patterns of body mass index milestones in early life and cardiometabolic risk in early adolescence. Int J Epidemiol 2019;48:157–167. 30624710 10.1093/ije/dyy286 PMC6380298

[deab164-B3] Woo Baidal JA , Locks LM, Cheng ER, Blake-Lamb TL, Perkins ME, Taveras EM. Risk factors for childhood obesity in the first 1,000 days: a systematic review. Am J Prev Med 2016;50:761–779. 26916261 10.1016/j.amepre.2015.11.012

[deab164-B4] Bleil ME , Appelhans BM, Adler NE, Gregorich SE, Sternfeld B, Cedars MI. Pubertal timing, androgens, and obesity phenotypes in women at midlife. J Clin Endocrinol Metab 2012;97:E1948–E1952. 22865899 10.1210/jc.2012-1972 PMC3462930

[deab164-B5] Boivin J , Bunting L, Collins JA, Nygren KG. International estimates of infertility prevalence and treatment-seeking: potential need and demand for infertility medical care. Hum Reprod 2007;22:1506–1512. 17376819 10.1093/humrep/dem046

[deab164-B6] Brewer CJ , Balen AH. The adverse effects of obesity on conception and implantation. Reproduction 2010;140:347–364. 20395425 10.1530/REP-09-0568

[deab164-B7] Bulik CM , Sullivan PF, Fear JL, Pickering A, Dawn A, McCullin M. Fertility and reproduction in women with anorexia nervosa: a controlled study. J Clin Psychiatry 1999;60:130–137. 10084645 10.4088/jcp.v60n0212

[deab164-B8] Carroll J , Saxena R, Welt CK. Environmental and genetic factors influence age at menarche in women with polycystic ovary syndrome. J Pediatr Endocrinol Metab 2012;25:459–466. 22876539 10.1515/jpem-2012-0047 PMC3597236

[deab164-B9] Chen J , Zhong C, Liang H, Yang Y, Zhang O, Gao E, Chen A, Yuan W, Wang J, Sun F. et al The relationship between age at menarche and infertility among Chinese rural women. Eur J Obstet Gynecol Reprod Biol 2015;194:68–72. 26334357 10.1016/j.ejogrb.2015.08.016

[deab164-B10] Couto Alves A , De Silva NMG, Karhunen V, Sovio U, Das S, Taal HR, Warrington NM, Lewin AM, Kaakinen M, Cousminer DL. et al GWAS on longitudinal growth traits reveals different genetic factors influencing infant, child, and adult BMI. Sci AdvAdv. Early Growth Genetics (EGG) Consortium. 2019;5:eaaw3095. 10.1126/sciadv.aaw3095 PMC6904961 31840077

[deab164-B11] DeKeyser N , Josefsson A, Bladh M, Carstensen J, Finnström O, Sydsjö G. Premature birth and low birthweight are associated with a lower rate of reproduction in adulthood: a Swedish population–based registry study. Hum Reprod 2012;27:1170–1178. 22286265 10.1093/humrep/der478

[deab164-B12] Easter A , Treasure J, Micali N. Fertility and prenatal attitudes towards pregnancy in women with eating disorders: results from the Avon Longitudinal Study of Parents and Children. BJOG Int J Obstet Gynaecol 2011;118:1491–1498. 10.1111/j.1471-0528.2011.03077.x 21810162

[deab164-B13] Ekholm K , Carstensen J, Finnström O, Sydsjö G. The probability of giving birth among women who were born preterm or with impaired fetal growth: a Swedish population-based registry study. Am J Epidemiol 2005;161:725–733. 15800264 10.1093/aje/kwi096

[deab164-B14] Euling SY , Selevan SG, Pescovitz OH, Skakkebaek NE. Role of environmental factors in the timing of puberty. Pediatrics 2008;121:S167–S172. 18245510 10.1542/peds.2007-1813C

[deab164-B15] Forest MG , Cathiard AM, Bertrand JA. Evidence of testicular activity in early infancy. J Clin Endocrinol Metab 1973;37:148–151. 4715291 10.1210/jcem-37-1-148

[deab164-B9462080] Gunderson EP , Murtaugh MA, Lewis CE, Quesenberry CP, West DS, Sidney S. Excess gains in weight and waist circumference associated with childbearing: The Coronary Artery Risk Development in Young Adults Study (CARDIA). Int J Obes Relat Metab Disord 2004;28:525–535. 14770188 10.1038/sj.ijo.0802551 PMC3133634

[deab164-B16] He Y , Tian J, Oddy WH, Dwyer T, Venn AJ. Association of childhood obesity with female infertility in adulthood: a 25-year follow-up study. Fertil Steril 2018;110:596–604. 30196944 10.1016/j.fertnstert.2018.05.011

[deab164-B17] Herbert D , Lucke J, Dobson A. Agreement between self-reported use of in vitro fertilization or ovulation induction, and medical insurance claims in Australian women aged 28–36 years. Hum Reprod 2012;27:2823–2828. 22740497 10.1093/humrep/des228

[deab164-B18] Hughes AR , Sherriff A, Ness AR, Reilly JJ. Timing of adiposity rebound and adiposity in adolescence. Pediatrics 2014;134:e1354–e1361. 25311600 10.1542/peds.2014-1908

[deab164-B19] Jacobs MB , Bazzano LA, Pridjian G, Harville EW. Childhood adiposity and fertility difficulties: the Bogalusa Heart Study. Pediatr Obes 2017;12:477–484. 27350648 10.1111/ijpo.12168 PMC5589511

[deab164-B20] Jokela M , Kivimäki M, Elovainio M, Viikari J, Raitakari OT, Keltikangas-Järvinen L. Body Mass Index in adolescence and number of children in adulthood. Epidemiology 2007;18:599–606. 17700249 10.1097/EDE.0b013e3181257158

[deab164-B21] Karjula S , Morin-Papunen L, Auvinen J, Ruokonen A, Puukka K, Franks S, Jarvelin M-R, Tapanainen JS, Jokelainen J, Miettunen J. et al Psychological distress is more prevalent in fertile age and premenopausal women with PCOS symptoms: 15-year follow-up. J Clin Endocrinol Metab 2017;102:1861–1869. 28323926 10.1210/jc.2016-3863 PMC5470769

[deab164-B22] Kiviranta P , Kuiri-Ha Nninen T, Saari A, Lamidi M-L, Dunkel L, Sankilampi U. Transient postnatal gonadal activation and growth velocity in infancy. Pediatrics 2016;138:e20153561. 27283013 10.1542/peds.2015-3561

[deab164-B23] Koivuaho E , Laru J, Ojaniemi M, Puukka K, Kettunen J, Tapanainen JS, Franks S, Järvelin M-R, Morin-Papunen L, Sebert S. et al Age at adiposity rebound in childhood is associated with PCOS diagnosis and obesity in adulthood-longitudinal analysis of BMI data from birth to age 46 in cases of PCOS. Int J Obes 2019;43:1370–1379. 10.1038/s41366-019-0318-z PMC6760596 30718819

[deab164-B24] Koivunen R , Pouta A, Franks S, Martikainen H, Sovio U, Hartikainen A, McCarthy MI, Ruokonen A, Bloigu A, Järvelin MR. et al Fecundability and spontaneous abortions in women with self-reported oligo-amenorrhea and/or hirsutism: Northern Finland Birth Cohort 1966 study. Hum Reprod 2008;23:2134–2139. 18544581 10.1093/humrep/den136

[deab164-B25] Koyama S , Ichikawa G, Kojima M, Shimura N, Sairenchi T, Arisaka O. Adiposity rebound and the development of metabolic syndrome. Pediatrics 2014;133:e114–e119. 24366997 10.1542/peds.2013-0966

[deab164-B26] Kuczmarski RJ , Ogden CL, Grummer-Strawn LM, Flegal KM, Guo SS, Wei R, Mei Z, Curtin LR, Roche AF, Johnson CL. Advance Data for Vital and Health Statistics No. 314, 2000. Hyattsville, MD: National Center for Health Statistics. 11183293

[deab164-B27] Kuiri-Hänninen T , Sankilampi U, Dunkel L. Activation of the hypothalamic-pituitary-gonadal axis in infancy: minipuberty. Horm Res Paediatr 2014;82:73–80. 25012863 10.1159/000362414

[deab164-B28] Lake JK , Power C, Cole TJ. Women's reproductive health: the role of body mass index in early and adullife. Int J Obes Relat Metab Disord 1997;21:432–438. 9192225 10.1038/sj.ijo.0800424

[deab164-B29] Lanciotti L , Cofini M, Leonardi A, Penta L, Esposito S. Up-to-date review about minipuberty and overview on hypothalamic-pituitary-gonadal axis activation in fetal and neonatal life. Front Endocrinol 2018;9:410. 10.3389/fendo.2018.00410. PMC6070773 30093882

[deab164-B30] Lee JM , Appugliese D, Kaciroti N, Corwyn RF, Bradley RH, Lumeng JC. Weight status in young girls and the onset of puberty. Pediatrics 2007;119:e624–e630. 17332182 10.1542/peds.2006-2188

[deab164-B31] Maffeis C , Moghetti P, Grezzani A, Clementi M, Gaudino R, Tatò L. Insulin resistance and the persistence of obesity from childhood into adulthood. J Clin Endocrinol Metab 2002;87:71–76. 11788625 10.1210/jcem.87.1.8130

[deab164-B32] Moran A , Jacobs DR, Steinberger J, Hong CP, Prineas R, Luepker R, Sinaiko AR. Insulin resistance during puberty: results from clamp studies in 357 children. Diabetes 1999;48:2039–2044. 10512371 10.2337/diabetes.48.10.2039

[deab164-B33] Nelson SM , Fleming R. Obesity and reproduction: impact and interventions. Curr Opin Obstet Gynecol 2007;19:384–389. 17625423 10.1097/GCO.0b013e32825e1d70

[deab164-B34] Ollila M , Piltonen T, Puukka K, Ruokonen A, Järvelin MR, Tapanainen J, Franks S, Morin-Papunen L. Weight gain and dyslipidemia in early adulthood associate with polycystic ovary syndrome: prospective cohort study. J Clin Endocrinol Metab 2016;101:739–747. 26652764 10.1210/jc.2015-3543

[deab164-B35] Palmert MR , Gordon CM, Kartashov AI, Legro RS, Emans SJ, Dunaif A. Screening for abnormal glucose tolerance in adolescents with polycystic ovary syndrome. J Clin Endocrinol Metab 2002;87:1017–1023. 11889155 10.1210/jcem.87.3.8305

[deab164-B36] Péneau S , González-Carrascosa R, Gusto G, Goxe D, Lantieri O, Fezeu L, Hercberg S, Rolland-Cachera MF. Age at adiposity rebound: determinants and association with nutritional status and the metabolic syndrome at adulthood. Int J Obes 2016;40:1150–1156. 10.1038/ijo.2016.39 27113489

[deab164-B37] Perez RC. Current mapping of obesity. Nutr Hosp 2013;28(Suppl 5):21–31. 24010741 10.3305/nh.2013.28.sup5.6915

[deab164-B38] Pinola P , Lashen H, Bloigu A, Puukka K, Ulmanen M, Ruokonen A, Martikainen H, Pouta A, Franks S, Hartikainen AL. et al Menstrual disorders in adolescence: a marker for hyperandrogenaemia and increased metabolic risks in later life? Finnish general population-based birth cohort study. Hum Reprod 2012;27:3279–3286. 22933528 10.1093/humrep/des309

[deab164-B39] Rantakallio P. The longitudinal study of the Northern Finland birth cohort of 1966. Paediatr Perinat Epidemiol 1988;2:59–88. 2976931 10.1111/j.1365-3016.1988.tb00180.x

[deab164-B40] Rolland-Cachera MF , Deheeger M, Maillot M, Bellisle F. Early adiposity rebound: causes and consequences for obesity in children and adults. Int J Obes 2006;30:S11–S17. 10.1038/sj.ijo.0803514 17133230

[deab164-B41] Serdula MK , Ivery D, Coates RJ, Freedman DS, Williamson DF, Byers T. Do obese children become obese adults? A review of the literature. Prev Med 1993;22:167–177. 8483856 10.1006/pmed.1993.1014

[deab164-B42] Silverwood RJ , De Stavola BL, Cole TJ, Leon DA. BMI peak in infancy as a predictor for later BMI in the Uppsala Family Study. Int J Obes (Lond) 2009;33:929–937. 19564879 10.1038/ijo.2009.108

[deab164-B43] Simmonds M , Burch J, Llewellyn A, Griffiths C, Yang H, Owen C, Duffy S, Woolacott N. The use of measures of obesity in childhood for predicting obesity and the development of obesity-related diseases in adulthood: a systematic review and meta-analysis. Health Technol Assess 2015;19:1–336. 10.3310/hta19430 PMC4781104 26108433

[deab164-B44] Sovio U , Kaakinen M, Tzoulaki I, Das S, Ruokonen A, Pouta A, Hartikainen AL, Molitor J, Järvelin M-R. How do changes in body mass index in infancy and childhood associate with cardiometabolic profile in adulthood? Findings from the Northern Finland Birth Cohort 1966 Study. Int J Obes 2014;38:53–59. 10.1038/ijo.2013.165 24080793

[deab164-B45] Stern JE , McLain AC, Louis GMB, Luke B, Yeung EH. Accuracy of self-reported survey data on assisted reproductive technology treatment parameters and reproductive history. Am J Obstet Gynecol 2016;215:219.e1–291.e6. 26875948 10.1016/j.ajog.2016.02.010 PMC4967378

[deab164-B46] Tabler J , Utz RI, Geist C, Hanson HA, Smith KA. Variation in reproductive outcomes of women with histories of bulimia nervosa, anorexia nervosa, or eating disorder not otherwise specified relative to the general population and closest-aged sisters. Int J Eat Disord 2018;51:102–111. 29331083 10.1002/eat.22827 PMC6599590

[deab164-B47] Tanner JM , Whitehouse RH. Clinical longitudinal standards for height, weight, height velocity, weight velocity, and stages of puberty. Arch Dis Child 1976;51:170–179. 952550 10.1136/adc.51.3.170 PMC1545912

[deab164-B48] Taponen S , Ahonkallio S, Martikainen H, Koivunen R, Ruokonen A, Sovio U, Hartikainen AL, Pouta A, Laitinen J, King V. et al Prevalence of polycystic ovaries in women with self-reported symptoms of oligomenorrhoea and/or hirsutism: Northern Finland Birth Cohort 1966 Study. Hum Reprod 2004;19:1083–1088. 15044401 10.1093/humrep/deh214

[deab164-B49] Taponen S , Martikainen H, JäRvelin M-R, Laitinen J, Pouta A, Hartikainen A-L, Sovio U, McCarthy MI, Franks S, Ruokonen A. Hormonal profile of women with self-reported symptoms of oligomenorrhea and/or hirsutism: Northern Finland Birth Cohort 1966 study. J Clin Endocrinol Metab 2003;88:141–147. 12519843 10.1210/jc.2002-020982

[deab164-B50] Ward ZJ , Long MW, Resch SC, Giles CM, Cradock AL, Gortmaker SL. Simulation of growth trajectories of childhood obesity into adulthood. N Engl J Med 2017;377:2145–2153. 29171811 10.1056/NEJMoa1703860 PMC9036858

[deab164-B51] Weghofer A , Kim A, Barad DH, Gleicher N. Age at menarche: a predictor of diminished ovarian function? Fertil Steril 2013;100:1039–1043. 23809497 10.1016/j.fertnstert.2013.05.042

[deab164-B52] Williamson DF , Madans J, Pamuk E, Flegal KM, Kendrick JS, Serdula MK. A prospective study of childbearing and 10-year weight gain in US white women 25 to 45 years of age. Int J Obes Relat Metab Disord 1994;18:561–569. 7951478

[deab164-B53] Wise LA , Mikkelsen EM, Rothman KJ, Riis AH, Sørensen HT, Huybrechts KF, Hatch EE. A prospective cohort study of menstrual characteristics and time to pregnancy. Am J Epidemiol 2011;174:701–709. 21719742 10.1093/aje/kwr130 PMC3166706

[deab164-B54] Yakusheva O , Kapinos K, Weiss M. Maternal weight after childbirth versus aging-related weight changes. Womens Health Issues 2017;27:174–180. 28094117 10.1016/j.whi.2016.12.001

