# Supplementary material for: BMI in childhood and adolescence is associated with impaired reproductive function—a population-based cohort study from birth to age 50 years
Source: Hum Reprod. 2021 Aug 7;36(11):2948–61. doi: 10.1093/humrep/deab164 (PMC8643422; doi:10.1093/humrep/deab164)
Supplement: deab164_Supplementary_Table_S1 [file deab164_supplementary_table_s1.pdf]

Human Reproduction, pp. 1–1, 2021
doi:10.1093/humrep/deab164

human reproduction

SUPPLEMENTARY DATA

Supplementary Table SI **Distribution of BMI in infancy and early childhood.**

BMI	6 months	12 months	24 months	3 years	4 years	5 years	6 years
Below 5th pc (%)	2.3	0.8	1.0	1.3	1.3	2.2	2.9
5th–85th pc (%)	73.0	57.7	68.1	76.1	85.4	87.1	84.7
85th–95th pc (%)	15.4	21.4	17.0	14.1	8.0	6.2	8.2
Over 95th pc (%)	9.3	20.1	13.9	8.6	5.2	4.5	4.2
Total number	2928	3797	1773	1633	1553	1428	1888

Classification has been made according to the World Health Organization (WHO) criteria (Center for Disease Control and Prevention, National Center for Health Statistics). Women who reported to have never attempted to achieve pregnancy were excluded from all analyses.
pc, percentile.
