# Supplementary material for: BMI in childhood and adolescence is associated with impaired reproductive function—a population-based cohort study from birth to age 50 years
Source: Hum Reprod. 2021 Aug 7;36(11):2948–61. doi: 10.1093/humrep/deab164 (PMC8643422; doi:10.1093/humrep/deab164)
Supplement: deab164_Supplementary_Table_S2 [file deab164_supplementary_table_s2.pdf]

Human Reproduction, pp. 1–1, 2021
doi:10.1093/humrep/deab164

human reproduction

SUPPLEMENTARY DATA

Supplementary Table SII Association between infancy underweight (BMI under 5th percentile (pc)) and 'obesity' (BMI over 95th (pc)) at ages 6 and 12 months and fertility outcomes compared to normal weight group (5th–95th pc).

		No infertility problems	Decreased fecundability at age 31 years	Infertility assessments before age 46 years	Infertility treatments before age 46 years	Childlessness at age 50 years
Age 6 months	Total number	1588	241	383	294	397
BMI < 5th pc	Prevalence (%)	2.0	2.1	2.1	2.7	3.3
	OR (CI95%) Crude	ref	1.08 (0.42–2.81)	1.12 (0.51–2.47)	1.49 (0.68–3.27)	1.54 (0.77–3.11)
	Model I	ref	1.08 (0.42–2.81)	1.12 (0.51–2.48)	1.43 (0.67–3.30)	1.39(0.68–3.02)
	Model II	ref	1.08 (0.41–2.86)	1.15 (0.52–2.57)	1.53 (0.68–3.43)	1.35 (0.63–2.92)
BMI > 95th pc	Prevalence (%)	8.7	10.0	11.0	10.9	8.1
	OR (CI95%) Crude	ref	1.16 (0.73–1.85)	1.15(0.77–1.70)	1.17 (0.75–1.81)	0.92 (0.60–1.40)
	Model I	ref	1.14 (0.72–1.83)	1.15 (0.76–1.69)	1.16 (0.74–1.83)	0.96 (0.61–1.45)
	Model II	ref	1.12 (0.69–1.80)	1.14(0.77–1.69)	1.16 (0.74–1.82)	0.98 (0.62–1.55)
Age 12 months	Total number	2067	312	490	390	530
BMI < 5th pc	Prevalence (%)	0.8	0.6	0.6	0.3	0.8
	OR (CI95%) Crude	ref	0.87 (0.45–1.47)	0.74 (0.22–2.54)	0.23 (0.04–2.36)	1.02 (0.78–1.31)
	Model I	ref	0.88 (0.43–1.49)	0.77 (0.23–2.78)	0.30 (0.04–2.40)	1.00 (0.77–1.31)
	Model II	ref	0.88 (0.43–1.50)	0.86 (0.25–2.96)	0.36 (0.05–2.78)	1.00 (0.76–1.31)
BMI > 95th pc	Prevalence (%)	20.4	17.9	17.8	18.5	19.1
	OR (CI95%) Crude	ref	0.87 (0.63–1.19)	0.85 (0.65–1.11)	0.89 (0.68—1.19)	1.02 (0.80–1.31)
	Model I	ref	0.85 (0.61–1.18)	0.84 (0.60–1.10)	0.87 (0.65–1.16)	1.00 (0.76–1.31)
	Model II	ref	0.81 (0.59–1.11)	0.81 (0.61–1.06)	0.86 (0.64–1.15)	1.00 (0.76–1.30)

Women who reported to have never attempted to achieve pregnancy were excluded from the analyses.
Results are shown as odds ratios (OR) with 95% CI.
Model I: adjustment for marital status during reproductive period.
Model II: Model I + adjustment education and smoking at age 31 years.
