# Supplementary material for: BMI in childhood and adolescence is associated with impaired reproductive function—a population-based cohort study from birth to age 50 years
Source: Hum Reprod. 2021 Aug 7;36(11):2948–61. doi: 10.1093/humrep/deab164 (PMC8643422; doi:10.1093/humrep/deab164)
Supplement: deab164_Supplementary_Table_S3 [file deab164_supplementary_table_s3.pdf]

Human Reproduction, pp. 1–2, 2021
doi:10.1093/humrep/deab164

human reproduction

SUPPLEMENTARY DATA

Supplementary Table SIII Association between age and BMI at adiposity peak/rebound and between weight classes in different age groups and fertility outcomes.

		Decreased fecundability at age 31 years	Infertility assessments before age 46 years	Infertility treatments before age 46 years	Childlessness at age 50 years
**Adiposity peak OR 95% CI**					
Age (years)	Crude	0.88 (0.57–1.36)	0.86 (0.59–1.24)	0.91 (0.61–1.36)	1.01 (0.73–1.39)
	Model I	0.89 (0.58–1.37)	0.86 (0.59–1.24)	0.91 (0.61–1.36)	1.00 (0.70–1.42)
	Model II	0.86 (0.57–1.37)	0.86 (0.60–1.24)	0.91 (0.61–1.36)	0.98 (0.70–1.41)
BMI (kg/m2)	Crude	1.08 (0.86–1.34)	0.88 (0.73–1.06)	0.87 (0.71–1.08)	**0.84 (0.70–0.99)**
	Model I	1.08 (0.86–1.34)	0.88 (0.74–1.06)	0.88 (0.71–1.08)	0.83 (0.69–1.00)
	Model II	1.08 (0.87–1.35)	0.89 (0.74–1.07)	0.88 (0.71–1.08)	0.83 (0.69–1.01)
**Adiposity rebound OR 95% CI**					
Age (years)	Crude	0.86 (0.72–1.04)	1.06 (0.91–1.24)	1.09 (0.92–1.29)	0.89 (0.76–1.02)
	Model I	0.87 (0.72–1.04)	1.06 (0.91–1.24)	1.10 (0.93–1.31)	**0.86 (0.73–0.99)**
	Model II	0.87 (0.72–1.05)	1.07 (0.91–1.25)	1.09 (0.92–1.31)	0.86 (0.74–1.00)
BMI (kg/m2)	Crude	1.10 (0.95–1.27)	**0.87 (0.76–0.99)**	0.88 (0.76–1.01)	1.01 (0.89–1.14)
	Model I	1.10 (0.95–1.27)	**0.87 (0.77–0.99)**	0.87 (0.76–1.01)	0.99 (0.87–1.13)
	Model II	1.10 (0.95–1.27)	**0.87 (0.76–0.98)**	0.88 (0.75–1.01)	0.99 (0.87–1.12)
**Age 3–6 years OR 95% CI**					
Underweight	Crude	1.12 (0.50–2.51)	1.45 (0.81–2.62)	1.39 (0.71–2.70)	1.23 (0.67–2.25)
	Model I	1.10 (0.49–2.47)	1.42 (0.80–2.59)	1.36 (0.69–2.65)	1.55 (0.83–2.89)
	Model II	1.16 (0.51–2.61)	1.48 (0.81–2.64)	1.39 (0.71–2.72)	1.59 (0.85–2.98)
Overweight	Crude	0.99 (0.64–1.52)	0.84 (0.58–1.21)	0.83 (0.55–1.26)	0.83 (0.55–1.26)
	Model I	0.98 (0.64–1.52)	0.84 (0.58–1.21)	0.83 (0.55–1.25)	0.83 (0.55–1.25)
	Model II	0.99 (0.64–1.53)	0.84 (0.58–1.22)	0.83 (0.55–1.26)	0.83 (0.55–1.26)
Obese	Crude	1.25 (0.73–2.41)	0.65 (0.38–1.14)	0.67 (0.37–1.25)	0.82 (0.50–1.34)
	Model I	1.23 (0.72–2.12)	0.65 (0.37–1.13)	0.67 (0.36–1.24)	0.74 (0.43–1.26)
	Model II	1.24 (0.72–2.13)	0.64 (0.36–1.11)	0.66 (0.35–1.22)	0.74 (0.43–1.25)
**Age 7–10 years OR 95% CI**					
Underweight	Crude	0.86 (0.44–1.69)	1.37 (0.86–2.16)	1.31 (0.79–2.18)	1.03 (0.63–1.68)
	Model I	0.86 (0.44–1.69)	1.35 (0.86–2.14)	1.30 (0.78–2.16)	1.00 (0.69–1.70)
	Model II	0.84 (0.46–1.65)	1.37 (0.87–2.18)	1.31 (0.78–2.18)	1.00 (0.59–1.71)
Overweight	Crude	1.28 (0.81–2.05)	1.13 (0.77–1.67)	1.10 (0.71–1.68)	1.23 (0.85–1.78)
	Model I	1.28 (0.80–2.04)	1.12 (0.76–1.65)	1.07 (0.70–1.65)	1.35 (0.91–2.00)
	Model II	1.28 (0.80–2.04)	1.11 (0.75–1.64)	1.08 (0.70–1.66)	1.31 (0.88–1.94)
Obese	Crude	**1.88 (1.08–3.27)**	0.71 (0.37–1.35)	0.73 (0.36–1.47)	1.18 (0.70–1.98)
	Model I	**1.87 (1.07–3.24)**	0.70 (0.37–1.35)	0.71 (0.35–1.44)	1.00 (0.56–1.77)
	Model II	**1.86 (1.07–3.28)**	0.71 (0.37–1.35)	0.73 (0.36–1.47)	0.99 (0.56–1.77)
**Age 11–15 years OR 95% CI**					
Underweight	Crude	0.92 (0.53–1.58)	1.42 (0.97–2.09)	**1.54 (1.02–2.33)**	1.14 (0.76–1.71)
	Model I	0.92 (0.53–1.58)	1.42 (0.97–2.09)	**1.55 (1.02–2.34)**	1.25 (0.82–1.93)
	Model II	0.89 (0.51–1.53)	1.43 (0.97–2.10)	**1.55 (1.02–2.36)**	1.27 (0.82–1.95)
Overweight	Crude	1.16 (0.74–1.82)	0.87 (0.58–1.31)	0.93 (0.60–1.46)	1.68 (0.96–1.96)
	Model I	1.16 (0.74–1.83)	0.86 (0.57–1.32)	0.91 (0.58–1.42)	**1.57 (1.08–2.29)**

(continued)

Supplementary Table SIII Continued

		Decreased fecundability at age 31 years	Infertility assessments before age 46 years	Infertility treatments before age 46 years	Childlessness at age 50 years
	Model II	1.04 (0.63–1.73)	0.86 (0.57–1.30)	0.94 (0.59–1.45)	**1.56 (1.06–2.27)**
Obese	Crude	1.78 (0.97–3.23)	1.21 (0.68–2.16)	1.13 (0.59–2.18)	**1.68 (1.01–2.79)**
	Model I	1.75 (0.97–3.21)	1.21 (0.68–2.15)	1.11 (0.57–2.14)	**1.77 (1.02–3.01)**
	Model II	1.82 (0.99–3.33)	1.22 (0.68–2.18)	1.16 (0.60–2.24)	**1.77 (1.02–3.07)**

Normal weight is used as a reference group.
Women with polycystic ovary syndrome were excluded. Women who reported to have never attempted to achieve pregnant were excluded from the analyses.
Underweight (BMI <5th percentile (pc)), normal weight (BMI 5th–85th pc), overweight (BMI 85th–85th pc), obese (BMI >95th pc).
Results are shown as OR with 95% CI.
Model I: adjustment for marital status during reproductive period.
Model II: Model I + adjustment for education and smoking.
The bold values significance is expressed in the Tables as ORs.
